# Slowing Thoracic Aortic Aneurysm Growth with Statins: A Meta-Analysis

**DOI:** 10.2174/011573403X343512250127075044

**Published:** 2025-02-13

**Authors:** Amie Marie Kolimas, Gargya Malla, Abhimanyu Chadha, Enkhtsogt Sainbayar, Joshua Sethi, Ziad Hindosh, Priyanka Hadvani, Hoang Nhat Pham, Juan Sordia

**Affiliations:** 1 Department of Medicine, University of Arizona, Tucson, AZ 85721, United States;; 2 Department of Cardiology, Honor Health, Phoenix, 1625 N Campbell Ave, Tucson, AZ 85719, United States

**Keywords:** Thoracic aorta, aneurysm, thoracic aortic aneurysms, statins, heterogeneity, metalloproteases, data analysis

## Abstract

**Introduction:**

Thoracic aortic aneurysms (TAAs) are worrisome for their propensity to dissect. Previous studies have demonstrated the potential benefits of statin use, particularly with slowing aortic aneurysm growth. The aim of this meta-analysis was to consolidate existing research to ascertain if statins effectively reduce TAA growth.

**Methods:**

Multiple databases were searched to identify studies assessing TAA growth in patients on statins (cases) and those not on statins (controls). The primary outcome was TAA (ascending/aortic arch) growth rate per year. Standard mean difference (SMD) and 95% confidence intervals (95% CI) were estimated with a random-effects model using the inverse-variance technique. We assigned I2>50% as an indicator of statistical heterogeneity. *P-*value <0.05 was considered significant. Data analysis was performed using SPSS v.25.0.

**Results:**

Four studies comprising 757 cases (male 64%, mean age 65±14 years) and 1,696 controls (male 62%, mean age 61±18 years) were included. The baseline diameters of TAA for cases and controls were 40.35±8.75 mm and 42.39±12.60 mm, respectively. Pooled results suggested statins to be associated with slower growth of TAAs with pooled SMD -0.70 mm/year [95% CI (-1.23 – -0.16); *p=*0.01]. Heterogeneity statistics among 4 studies was 95%.

**Conclusion:**

This pooled meta-analysis showed statins as associated with slower growth of TAAs. However, given the heterogeneity of the included studies in this meta-analysis, results should be interpreted with caution.

## 1 INTRODUCTION

Thoracic aortic aneurysms (TAAs) have an incidence of 5 to 10 cases per 100,000 individuals [1, 2]. A TAA is characterized by the abnormal dilation of the aorta, enlarging it to at least 1.5 times its normal parameters [3, 4]. This condition often remains asymptomatic until a life-threatening complication occurs, such as a rupture or dissection [5, 6]. Notably, an aneurysm exceeding 6 centimeters in diameter has been associated with an annual rupture or dissection rate of 6.9% and a mortality rate of 11.8% [7-9]. Current guidelines address the treatment of those with symptomatic TAAs through either open surgical repair or thoracic endovascular aortic repair (TEVAR) [10, 11].

A well-known precursor to TAA and eventual aortic dissection and rupture is a larger baseline aortic diameter, which is often associated with male sex, age, height, weight, and other traditional cardiovascular risk factors [12]. Notably, aortic dissections frequently manifest before the aortic diameter reaches the surgical threshold for preventive intervention [13]. As a result, it is essential to integrate lifestyle modifications and pharmacological interventions into the preventive management of TAAs to prevent life-threatening complications associated with these aneurysms. Studies have suggested that statins could have a notable impact on aneurysm development and progression by influencing metalloproteases and cardiac wall remodeling [14]. Therefore, the use of statin therapy may offer the potential to lower TAA-related mortality and improve long-term patient outcomes [15, 16]. In this meta-analysis, we aimed to analyze available data to assess the efficacy of statins on TAA growth.

## 2 METHODS

The meta-analysis was performed following the Preferred Reporting Items for Systematic Review and Meta-analysis (PRISMA) guidelines [17]. A literature search was conducted across 5 databases (PubMed, Scopus, Embase, Google Scholar, Cochrane CENTRAL database) to collect all articles reporting data on the effect of statins on the growth of the ascending aorta and aortic arch. The following search terms included statin, statins, atorvastatin, rosuvastatin, pravastatin, simvastatin, pitavastatin, fluvastatin, lovastatin, thoracic/thoracoabdominal aortic/aorta aneurysm/dilation. Additionally, manual hand-searching was also performed on reference lists of the included studies, review articles, and previous meta-analyses. All databases were searched from their inception to the date 09/17/2023.

Data screening and extraction were conducted by two independent authors. Disagreements were resolved by consulting a third independent author as needed. Initial screening of retrieved articles was completed by assessing titles and abstracts using Covidence (https://www.covidence.org), a web-based tool developed for systematic reviews. If appropriate, full-text screening of the selected articles was performed based on our stringent inclusion and exclusion criteria. Inclusion criteria comprised any observational study or randomized clinical trials assessing ascending aorta and arch aneurysm growth in patients taking (cases) or not taking statins (controls). Exclusion criteria included articles without available full text, conference abstracts, or posters. No restrictions on age, sex, race, ethnicity, language, country, or publication date were imposed. A standardized extraction sheet was used for data extraction. Data extraction included study title, year of publication, study design, number of participants for both cases and controls, baseline characteristics, baseline TAA diameters, and primary outcome (aortic aneurysm growth rate via centimeter per year).

The SPSS software version 25.0 was used to perform the meta-analysis and create the forest plot. Standard mean difference (SMD) and 95% confidence interval (95% CI) were estimated with a random-effects model using the inverse-variance technique. We assigned I2>50% as an indicator of statistical heterogeneity. *P-*value <0.05 was considered significant.

## 3 RESULTS

**Fig. (1):**
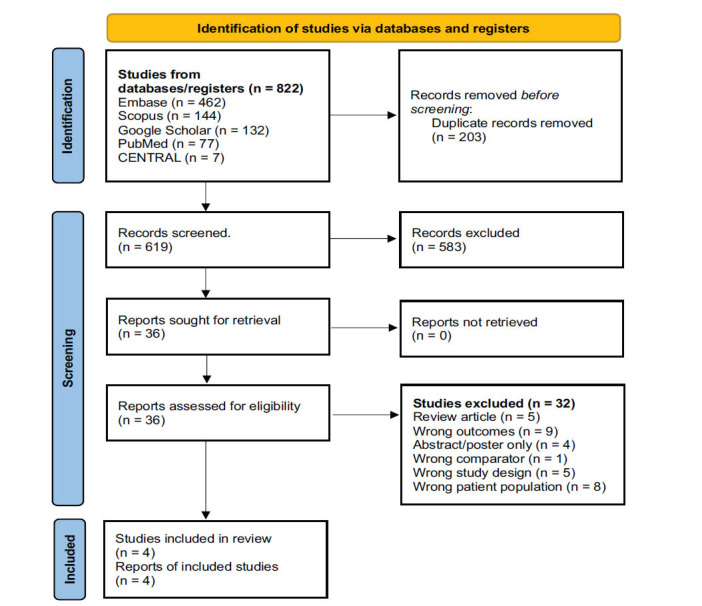
Flowchart demonstrating the study filtration process.

A total of 4 studies were included in our meta-analysis (Fig. **1**) [18-21]. We included a total of 757 patients on statins (male 64%, mean age 65±14 years) and 1,696 control patients (male 62%, mean age 61±18 years). The baseline diameters of TAA for cases and controls were 40.35 ± 8.75mm and 42.39 ± 12.60 mm, respectively. A description of the four included studies is presented in Table **1**.

**Fig. (2):**
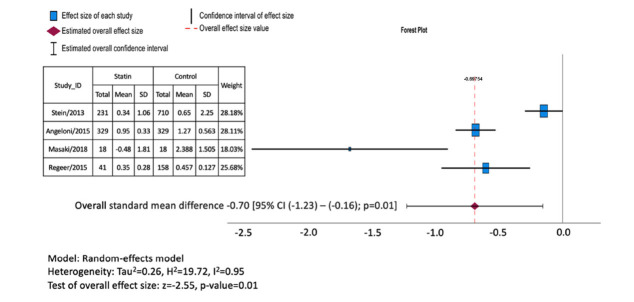
Forest plot: the effect of statin on thoracic aortic aneurysm growth.

Pooled results suggested statins to be associated with slower growth of TAAs with a pooled standard mean difference of -0.70 mm/year [95% CI (-1.23 – -0.16); *p=*0.0^1^] (Fig. **2**). Despite the heterogeneity with the *I^2^* value of 95%, we managed potential biases by utilizing a random effects model through the inverse variance technique.

## 4 DISCUSSION

While surgical intervention is often a primary component in the management of TAAs, emerging data suggest that statins may have a role in slowing the progression of TAAs [5, 22, 23]. This meta-analysis, encompassing four studies with a collective cohort of 2,453 patients, investigated the impact of statin use against non-usage on TAA progression. The pooled results demonstrated statin use as associated with a decelerated rate of TAA expansion.

Studies have elucidated that statins may have a role in the management of aortic aneurysms and have demonstrated improved long-term outcomes, particularly in reducing the progression of the aneurysm as well as reducing the odds of requiring surgery [15, 24]. This was also reported to be associated with reduced mortality with statin use compared to non-use in this patient population [25]. The exact mechanism of statins slowing the progression of the aneurysm is not entirely clear, but some possible mechanisms have been elucidated. Previous studies have shown that histologically, aneurysm pathogenesis is associated with inflammation, vascular smooth muscle apoptosis, extracellular matrix degradation, and arterial remodeling [26-30]. Statins have demonstrated the ability to suppress or inhibit various inflammatory processes in aneurysmal walls [31-33]. In addition to their anti-inflammatory effects, statins have also been shown to promote antioxidant effects. Through mitigation of oxidative stress within the aortic wall, statins can protect against cellular damage that may lead to the progression of TAAs [34-37]. Furthermore, statins have also been reported to preserve certain elastic proteins and vascular smooth muscle integrity. Disruptions in these structural components are implicated in the progression of TAAs. Therefore, statins can promote the integrity of the aortic wall by preserving these crucial vascular elements [38-40]. This meta-analysis was constrained by the inherent heterogeneity observed among the included studies. Nevertheless, a random-effects model was employed to mitigate this limitation.

This meta-analysis faced several limitations, one of which was the heterogeneity observed in the study sample. This variability may have stemmed from differences in the study populations, geographic locations, and the specific statin regimens employed across individual studies. Another limitation was the small sample size as the analysis included data from only four studies.

## CONCLUSION

This pooled meta-analysis revealed statins to be associated with slower growth of TAAs. Their influence on anti-inflammatory mediators, antioxidant effects, and the ability to preserve aortic wall integrity suggest their potential role in slowing TAA progression. Further research into the comprehensive therapeutic potential of statins is warranted to enhance the management and subsequent outcomes of this challenging cardiovascular condition. Additionally, it is important to evaluate whether the heterogeneity of the studies has potentially influenced the study findings, emphasizing the need for future research that closely examines patient characteristics and therapies utilized in the analyzed cohort.

## Figures and Tables

**Table 1:** Description of included studies.

**Study**	**Type of Study**	**No. of Patients**	**Aneurysm Location**	**Statin Used**
Stein, 2013	Retrospective, observational	941 (S=231; C=710)	Thoracic	Unspecified
Angeloni, 2015	Prospective, randomized	658 (S=329; C=329)	Thoracic	Simvastatin, rosuvastatin, atorvastatin, lovastatin
Masaki, 2018	Prospective, randomized	36 (S=18; C=18)	Thoracic	Pitavastatin
Regeer, 2015	Retrospective, observational	199 (S=41; C=158)	Thoracic	Simvastatin, rosuvastatin, atorvastatin, pravastatin

**Abbreviations:** S = Statin therapy group, C - Control group.

## Data Availability

All data generated or analyzed during this study are included in this published article.

## LIST OF ABBREVIATIONS

SMD: Standard Mean Difference
TAAs: Thoracic Aortic Aneurysms
TEVAR: Thoracic Endovascular Aortic Repair

## STANDARDS OF REPORTING

PRISMA guidelines were followed.

## SUPPLEMENTARY MATERIAL

PRISMA checklist as available on the publisher's website along with the published article.
